# Rapid bacterial colonization of low-density polyethylene microplastics in coastal sediment microcosms

**DOI:** 10.1186/s12866-014-0232-4

**Published:** 2014-09-23

**Authors:** Jesse P Harrison, Michaela Schratzberger, Melanie Sapp, A Mark Osborn

**Affiliations:** Department of Animal and Plant Sciences, The University of Sheffield, Sheffield, S10 2TN, UK; The Centre for Environment, Fisheries and Aquaculture Science, Lowestoft, NR33 0HT, UK; Department of Biological Sciences, University of Hull, Kingston-upon-Hull, HU6 7RX, UK; Current address: School of Physics and Astronomy, The University of Edinburgh, Edinburgh, EH9 3JZ, UK; Current address: The Food and Environment Research Agency, Sand Hutton York, YO41 1LZ, UK; Current address: School of Applied Sciences, RMIT University, Bundoora, Melbourne, VIC3083, Australia

**Keywords:** Bacteria, Sediment, Microplastics, Succession, *Arcobacter*, *Colwellia*

## Abstract

**Background:**

Synthetic microplastics (≤5-mm fragments) are emerging environmental contaminants that have been found to accumulate within coastal marine sediments worldwide. The ecological impacts and fate of microplastic debris are only beginning to be revealed, with previous research into these topics having primarily focused on higher organisms and/or pelagic environments. Despite recent research into plastic-associated microorganisms in seawater, the microbial colonization of microplastics in benthic habitats has not been studied. Therefore, we employed a 14-day microcosm experiment to investigate bacterial colonization of low-density polyethylene (LDPE) microplastics within three types of coastal marine sediment from Spurn Point, Humber Estuary, U.K.

**Results:**

Bacterial attachment onto LDPE within sediments was demonstrated by scanning electron microscopy and catalyzed reporter deposition fluorescence *in situ* hybridisation (CARD-FISH). Log-fold increases in the abundance of 16S rRNA genes from LDPE-associated bacteria occurred within 7 days with 16S rRNA gene numbers on LDPE surfaces differing significantly across sediment types, as shown by quantitative PCR. Terminal-restriction fragment length polymorphism (T-RFLP) analysis demonstrated rapid selection of LDPE-associated bacterial assemblages whose structure and composition differed significantly from those in surrounding sediments. Additionally, T-RFLP analysis revealed successional convergence of the LDPE-associated communities from the different sediments over the 14-day experiment. Sequencing of cloned 16S rRNA genes demonstrated that these communities were dominated after 14 days by the genera *Arcobacter* and *Colwellia* (totalling 84–93% of sequences). Attachment by *Colwellia* spp. onto LDPE within sediments was confirmed by CARD-FISH.

**Conclusions:**

These results demonstrate that bacteria within coastal marine sediments can rapidly colonize LDPE microplastics, with evidence for the successional formation of plastisphere-specific bacterial assemblages. Although the taxonomic compositions of these assemblages are likely to differ between marine sediments and the water column, both *Arcobacter* and *Colwellia* spp. have previously been affiliated with the degradation of hydrocarbon contaminants within low-temperature marine environments. Since hydrocarbon-degrading bacteria have also been discovered on plastic fragments in seawater, our data suggest that recruitment of hydrocarbonoclastic bacteria on microplastics is likely to represent a shared feature between both benthic and pelagic marine habitats.

**Electronic supplementary material:**

The online version of this article (doi:10.1186/s12866-014-0232-4) contains supplementary material, which is available to authorized users.

## Background

Plastic debris is globally distributed across estuarine and marine ecosystems [1-8], reflecting the success of synthetic polymers as both consumer and industrial products, and their persistence in the environment [9]. Annual worldwide manufacture of thermoplastics has increased from less than two million tonnes in 1950 to between 230 and 245 million tonnes during the last decade [10]. There is now widespread public concern about the ecological impacts of plastic waste on marine organisms. Whilst the physical impacts caused by plastic debris (for example, entanglement and suffocation of wildlife) are well-recognized [11], the rapid proliferation of microplastics (≤5 mm fragments) in marine habitats [2,8,12] is leading to a re-evaluation of the potential detrimental effects of plastic litter [13]. Microplastics represent both a physical and chemical threat to the ecological integrity of our seas and oceans [9] due to their high potential to become ingested by wildlife, and their capacity to transport persistent organic pollutants (for example, polynuclear aromatic hydrocarbons) and plastic additives into marine food webs [14,15]. This is particularly true for coastal and intertidal sediments that represent sinks for the accumulation of plastic litter [7,9,12,16-18], where microplastic concentrations may reach up to 81 parts per million by mass [16] and constitute as much as 80% of plastic debris within the seafloor [19].

Most previous research into the environmental impacts of microplastics has focused either on their abundance and distribution [8,9] or on their potential detrimental effects on higher organisms [20]. In marine environments, microorganisms function as pioneering surface colonizers and drive critical ecosystem processes including primary production, biogeochemical cycling and the biodegradation of anthropogenic pollutants [21,22]. However, ecological interactions between marine microorganisms and microplastics have until recently received scant attention, with our understanding of this topic being limited primarily to pelagic habitats [23]. Early observations of the microbial colonization of microplastics in seawater reported the isolation of ‘rod shaped Gram-negative bacteria’ from ~0.5 mm polystyrene spherules [14] and the presence of diatoms on plastic fragments in the Sargasso Sea [1]. Culture-based seawater microcosm studies have also demonstrated microbial attachment to polyethylene terephthalate [24] and polyethylene plastic bags [25]. Moreover, experiments using molecular fingerprinting and 16S rRNA gene clone sequencing analyses have identified *Roseobacter* spp. and other *Alphaproteobacteria* as key colonists of acryl, polyurethane, poly(methyl methacrylate) and polyvinylchloride surfaces, following up to 72-hour exposures of these materials in coastal waters [21,22,26]. Recently, phylogenetically diverse plastic-associated microbial assemblages (including *Bacillus* and *Vibrio* spp.) have also been discovered in the North Pacific Gyre and the North Atlantic Ocean [27,28]. Moreover, bacterial association with plastic surfaces has been reported in engineered ecosystems, including drinking water distribution systems [29,30]. Despite these initial reports, there remains a lack of knowledge concerning microbial colonization of microplastic debris within marine environments. In particular, there is an absence of any information concerning microbial-plastic interactions and colonization processes within marine sediments [23].

Hence, in this study, we have used a 14-day laboratory microcosm experiment to investigate the potential for microplastics to function as sites for attachment of naturally occurring bacterial assemblages within coastal marine sediments. This research aimed firstly to quantify attachment of naturally occurring bacteria onto plastic fragments within sediments, and secondly to investigate variation in the structure and diversity of plastic-colonizing bacterial assemblages over time and across both sandy and silty sediment types. Thirdly, this study aimed to identify the predominant bacterial genera attaching onto the plastic surfaces. Low-density polyethylene (LDPE) was chosen as the model polymer due to its importance both as the most widely produced polymer, accounting for 21% of global production, and as a widely documented component of marine plastic debris [7-10].

## Methods

### Sediment sampling and characterisation

Sediments were collected on the 25^th^ of April 2010 from three sampling sites (SP1, SP2 and WB) within the Humber Estuary, UK (Additional file 1: Figure S1). Samples were obtained at low tide from the surface top centimeter of the sediment. Sediments were stored either overnight in darkness at 4°C prior to use in laboratory microcosms, or at −20°C for sedimentological analysis. Samples for sedimentological analysis were dried at 105°C and analyzed for loss on ignition (LOI) (%) and particle size distribution (PSD). The LOI measurements were based on changes in mass following heating at 425°C for 18 hours. Sediment size fractions of <1 mm were analyzed for PSD using a LA-950 particle sizer (Horiba Instruments Ltd, Northampton, UK), using 0.1 M sodium hexametaphosphate as a dispersant [31]. The sediments were predominantly comprised of fine sand (SP1), medium sand (SP2) and silt (WB) (Additional file 2: Table S1).

### Sediment-LDPE microcosms

Sediments were homogenized by stirring and microcosms were established by weighing 5 – 7 ml of sediment into sterile, triple-vented 55 mm Petri dishes. Each individual microcosm was spiked with six fragments (5 × 5 × 1 mm) of LDPE (Goodfellow Cambridge Ltd, Huntingdon, UK) that had been sterilized with 70% ethanol. The sediments were submerged in sterile artificial seawater (ASW) (ZM Systems, Winchester, UK). The salinity of the ASW was based on *in situ* measurements (33 at SP1 and 30 at SP2 and WB) taken using a Portasal™ 8410A salinometer (Guildline, Smiths Falls, Canada). Microcosms were incubated in darkness at 4°C (based on water temperature at the sites at the time of sediment sampling). Sacrificial sampling of plastic fragments was performed in triplicate (i.e., three replicate Petri-dish microcosms were sampled at each time point for each of the three sediment types, with each individual microcosm containing six LDPE fragments, totalling nine microcosms per time point) at seven intervals [immediately (within < 2 minutes), at 6 hours and after 1, 2, 4, 7 and 14 days]. The plastics were suspended, rinsed in ASW and centrifuged for five seconds (up to 2038 × *g*) in order to detach loosely adhering materials from the plastic surfaces, while retaining LDPE-attached cells for further analysis. LDPE fragments were stored at −80°C for molecular analyses or fixed overnight in 2% (v/v) formaldehyde at 4°C for scanning electron microscopy and fluorescence microscopy. Following fixation, fragments were rinsed with ASW and 96% ethanol, and stored at −20°C. Sediment samples were taken for molecular analysis from microcosms sampled at four time intervals (immediately and after 4, 7 and 14 days), and stored at −80°C.

### Scanning electron microscopy

Following initial fixation using 2% (v/v) formaldehyde, LDPE fragments were secondarily fixed in 2.5% glutaraldehyde in 0.1 M sodium cacodylate buffer for two hours at room temperature. The fragments were washed twice for 30 minutes with 0.1 M sodium cacodylate buffer and post-fixed in 2% osmium tetroxide for one hour. Samples were dehydrated by a graded series of exposures to ethanol and hexamethyldisilazane [32]. The samples were coated with ~25 nm of gold using an Edwards S150B sputter coater. Images were obtained with an XL-20 scanning electron microscope (Philips/FEI, Cambridge, UK) at an accelerating voltage of 20 kV.

### Catalyzed reporter deposition fluorescence *in situ* hybridization (CARD-FISH)

LDPE-associated bacterial cells were visualized using an existing CARD-FISH protocol [33] in conjunction with universal bacterial oligonucleotide probes (EUB338 I-III) and a negative control probe (NON338) (Thermo Fisher Scientific or Biomers, Ulm, Germany) (Table 1). Hybridization conditions employed for the CARD-FISH analysis are listed in Table 1. Probes targeting the genera *Arcobacter* (ARC94) and *Colwellia* (PSA184) (Biomers, Germany) were also used (Table 1), following bacterial 16S rRNA gene sequencing analysis (details below). CARD-FISH was performed according to the manufacturer’s instructions using a TSA™ Cyanine 3 Tyramide Reagent Pack (Perkin-Elmer, Buckinghamshire, UK). Following hybridization, the LDPE fragments were counterstained by 4′,6-diamidino-2-phenylindole (DAPI) [33]. Bacteria were visualized using an Olympus IX71 epifluorescence microscope equipped with a 100×/NA 1.3 objective.

**Table 1:** **Oligonucleotide probes and hybridization conditions used for CARD**-**FISH analysis of bacteria attached to LDPE fragments**

**Probe**	**Nucleotide sequence** **(** **5**′ **–** **3**′**)**	**Probe target**	**%** **FA** ^**a**^	**°** **C** ^**b**^	**References**
NON338	ACT CCT ACG GGA GGC AGC	Negative control	55	35	[34]
EUB338 I	GCT GCC TCC CGT AGG AGT	Most Bacteria	55	35	[35]
EUB338 II	GCA GCC ACC CGT AGG TGT	Planctomycetales	55	35	[36]
EUB338 III	GCT GCC ACC CGT AGG TGT	Verrumicrobiales	55	35	[36]
ARC94	TGC GCC ACT TAG CTG ACA	*Arcobacter*	20	46	[37]
PSA184	CCC CTT TGG TCC GTA GAC	*Pseudoalteromonas*, *Colwellia*	30	31	[38]

^a^Per cent (v/v) formamide (FA) in hybridization buffer, based on [39] (NON338, EUB probes), [37] (ARC94) or [38] (PSA184).

^b^Hybridization temperature, based on [39] (NON338, EUB probes), [37] (ARC94) or a modification of [40] (PSA184).

The fragments were retrieved from microcosms following 14 days of exposure to sediment from sampling site SP2 (Humber Estuary, UK).

### DNA isolation and end-point PCR amplification of 16S rRNA genes

DNA was isolated from either six pooled LDPE fragments (total surface area of 4.2 cm^2^) or from 0.5 g of sediment using a Powersoil® DNA isolation kit (MO BIO, Carlsbad, CA), and eluted in either 50 μl or 100 μl of sterile nuclease-free water (LDPE and sediment, respectively) (Ambion, Austin, USA). PCR was used to amplify 16S rRNA gene sequences for the construction of a standard curve for quantitative real-time PCR (Q-PCR) analysis [41,42], T-RFLP analysis [43] and for the construction of 16S rRNA gene clone libraries.

For standard curve construction for Q-PCR, the universal bacterial primers EUB338 (5′- ACT CCT ACG GGA GGC AGC AG −3′) and EUB518 (5′- ATT ACC GCG GCT GCT GG −3′) were used [44]. Each PCR contained 1 μl of sediment template DNA, 1× PCR buffer containing 1.5 mM of MgCl_2_, 0.25 mM of each deoxyribonucleoside triphosphate (dNTP), 0.3 μM of each primer and 2.5 U of *Taq* polymerase (Qiagen, Crawley, UK) made up to a total volume of 25 μl with sterile nuclease-free water (Ambion). PCR cycling conditions were 94°C for 3 min, followed by 40 cycles of 94°C for 30 s, 50°C for 45 s and 72°C for 30 s and a final extension step at 72°C for 7 min. PCR products used for standard curve construction were purified using a QIAquick® gel extraction kit (Qiagen).

For T-RFLP fingerprinting, the primers FAM-63 F (5′- CAG GCC TAA CAC ATG CAA GTC −3′) [45] and 1389R (5′- ACG GGC GGT GTG TAC AAG −3′) [46] were used. For amplification of 16S rRNA genes prior to clone library construction, primers 27 F (5′- AGA GTT TGA TCC TGG CTC AG −3′) and 1492R (5′- TAC CTT GTT ACG ACT T −3′) were used [47]. For both T-RFLP fingerprinting and library construction, each PCR contained 1–2 μl of template DNA, 1× PCR buffer containing 1.5 mM of MgCl_2_, 2× Q Solution (Qiagen), 0.25 mM of each dNTP, 0.4 μM of each primer and 2.5 U of *Taq* polymerase (Qiagen) made up to a total volume of 50 μl with sterile nuclease-free water. For T-RFLP analyses, PCR cycling conditions were 94°C for 2 min, followed by 35 cycles of 94°C for 30 s, 57°C for 45 s, 72°C for 90 s and a final extension step at 72°C for 10 min. For library construction, PCR cycling conditions were 94°C for 2 min, followed by 35 cycles of 94°C for 1 min, 55°C for 45 s, 72°C for 2 min and a final extension step at 72°C for 10 min. PCR products were visualized following electrophoresis on 0.8% (w/v) agarose gels. PCR products used for T-RFLP analyses were purified using QIAquick columns (Qiagen).

### Quantitative real-time PCR (Q-PCR) amplification of 16S rRNA genes

Purified PCR products (~200 bp) obtained from DNA extracted from sediments from sites SP2 and WB (see above) were used to construct a standard curve to quantify 16S rRNA gene numbers on LDPE surfaces across the three sites, over a range of 1.0 × 10^6^ to 1.0 × 10^9^ amplicons of target DNA per mm^2^ of LDPE. Quantitative real-time PCR (Q-PCR) analysis was performed on a single assay plate [48], using DNA extracted from LDPE fragments. Each Q-PCR contained 1 μl of template DNA, 5× QuantiFast® SYBR® Green PCR Mastermix (Qiagen) [49] and 0.3 μM of the primers EUB338 and EUB518 [44], made up to a total volume of 25 μl with sterile nuclease-free water (Ambion). Primers and cycling conditions were as described for end-point PCR amplifications, with the exception of omitting the final extension step. No-template controls (NTCs) (*n* = 3) were included. Measurements were performed in triplicate for each sampling interval and sediment type, using a CFX96™ Real-Time PCR Detection System (Bio-Rad, Hemel Hempstead, UK) and quantified by CFX Manager™ software (Bio-Rad). Mean cycle threshold (*C*_t_) values (i.e., the number of cycles required for the fluorescence signal to exceed the background) were estimated [50]. PCR product specificity was confirmed by melting curve analysis [51].

### Terminal-restriction fragment length polymorphism (T-RFLP) analysis

Purified PCR products (5 – 10 μl) were digested with 20 U of AluI and 1× restriction enzyme buffer (Roche, Burgess Hill, UK) in a total volume of 15 μl at 37°C for three hours. Digestion products (5 μl) were desalted using 0.2 mM MgSO_4_●7H_2_O and 5 μl of glycogen (20 mg ml^−1^) (Bioline, London, UK) in 70% ethanol. Desalted digests (1 – 4 μl) were denatured with formamide containing 0.5% GeneScan™ 500 ROX™ internal size standard (Applied Biosystems, Warrington, UK) in a total volume of 10 μl and incubated at 94°C for 3 min prior to electrophoresis using an ABI 3730 PRISM® Genetic Analyzer (Applied Biosystems), with injection times of 5 or 10 s and an injection voltage of 2 kV. Capillary electrophoresis was conducted at 15 kV for 20 minutes.

### 16S rRNA gene clone library construction and sequencing

Purified PCR products were ligated into the pCR4®-TOPO® TA cloning vector and transformed into One Shot® chemically competent *Escherichia coli* TOP10 cells (Invitrogen, Paisley, UK). Transformants were selected on Luria-Bertani (LB) agar plates containing ampicillin (50 μg ml^−1^) and X-gal (80 μg ml^−1^). Insert DNA from white colonies was amplified using the vector primers T7 (5′- TAA TAC GAC TCA CTA TAG G-3′) and T3 (5′- AAT TAA CCC TCA CTA AAG G −3′). Each PCR contained 1 μl of overnight culture, 1× PCR buffer, 1.5 mM of MgCl_2_, 0.25 mM of each dNTP, 0.4 μM of each primer and 2.5 U of *Taq* polymerase (Bioline), and was made up to a final volume of 25 μl with sterile nuclease-free water (Ambion). The PCR was performed as described for the primers 27 F and 1492R, using 25 cycles. PCR products were purified using the SureClean PCR purification kit (Bioline). Sequencing analysis was performed using 0.5 μM of primer 27 F, a BigDye® Terminator v3.1 cycle sequencing kit and an ABI 3730 PRISM® Genetic Analyser (Applied Biosystems).

### Bioinformatics and multivariate analyses

T-RFLP profiles were analysed using Genemapper® software (version 3.7, Applied Biosystems). Sizes of terminal restriction fragments (T-RFs) (50–500 nt) were estimated using the Local Southern method [52]. Peaks represented by fluorescence intensities of <100 units were excluded from further analyses. T-RFLP profiles were aligned using the software T-Align (http://inismor.ucd.ie/~talign/) [53]. The relative abundance of each T-RF was calculated as a proportion (%) of the total peak area within each profile. Peaks with relative areas of <0.5% were excluded. Square root-transformed Bray-Curtis similarity matrices based on the T-RFLP data were analysed using the PRIMER statistical package (version 6.1.13) [54] for non-parametric multidimensional scaling ordinations, analyses of similarity (ANOSIM) [55] and Shannon’s diversity [56]. A two-way ANOSIM was performed with ‘time’ (of exposure of LDPE-microplastics within sediment) and ‘substrate type’ (sediments and LDPE microplastic) as factors. One-way ANOSIMs were conducted with ‘sediment type’ or ‘time’ (of exposure of LDPE microplastics within sediment) as the factors. One-way ANOVAs for comparing Shannon’s diversity were performed using the R statistical package (version 2.12.0) [57], with ‘exposure time’ as the factor.

DNA sequences of cloned 16S rRNA genes were edited using ChromasPro software (version 1.5, http://www.technelysium.com.au). Multiple alignments were constructed using ClustalW2 (http://www.ebi.ac.uk/Tools/msa/clustalw2/), with chimeric sequences excluded using Mallard (version 1.02) [58] and Bellerophon [59]. Alignments were inspected for anomalous reads and trimmed to a universal read length with Mothur (version 1.17.2) [60]. Taxonomic assignments were performed using the Ribosomal Database Project Classifier (version 6) [61]. Sequences were compared to the GenBank database using the Basic Local Alignment Search Tool for nucleotides (BLASTn) [62]. Neighbor-joining trees [63] were constructed using MEGA (version 5.03) [64]. Evolutionary distances were calculated using the Kimura 2-parameter method [65]. Rate variation among sites was modelled by a gamma distribution with tree-specific shape parameters, as based on the maximum-likelihood fits of different nucleotide substitution models. Positions containing gaps and missing data were eliminated. Confidence levels for the tree topology were assessed by bootstrap analysis (1000 replicates).

## Results

### Bacterial attachment onto LDPE microplastics within sediments

**Figure 1:**
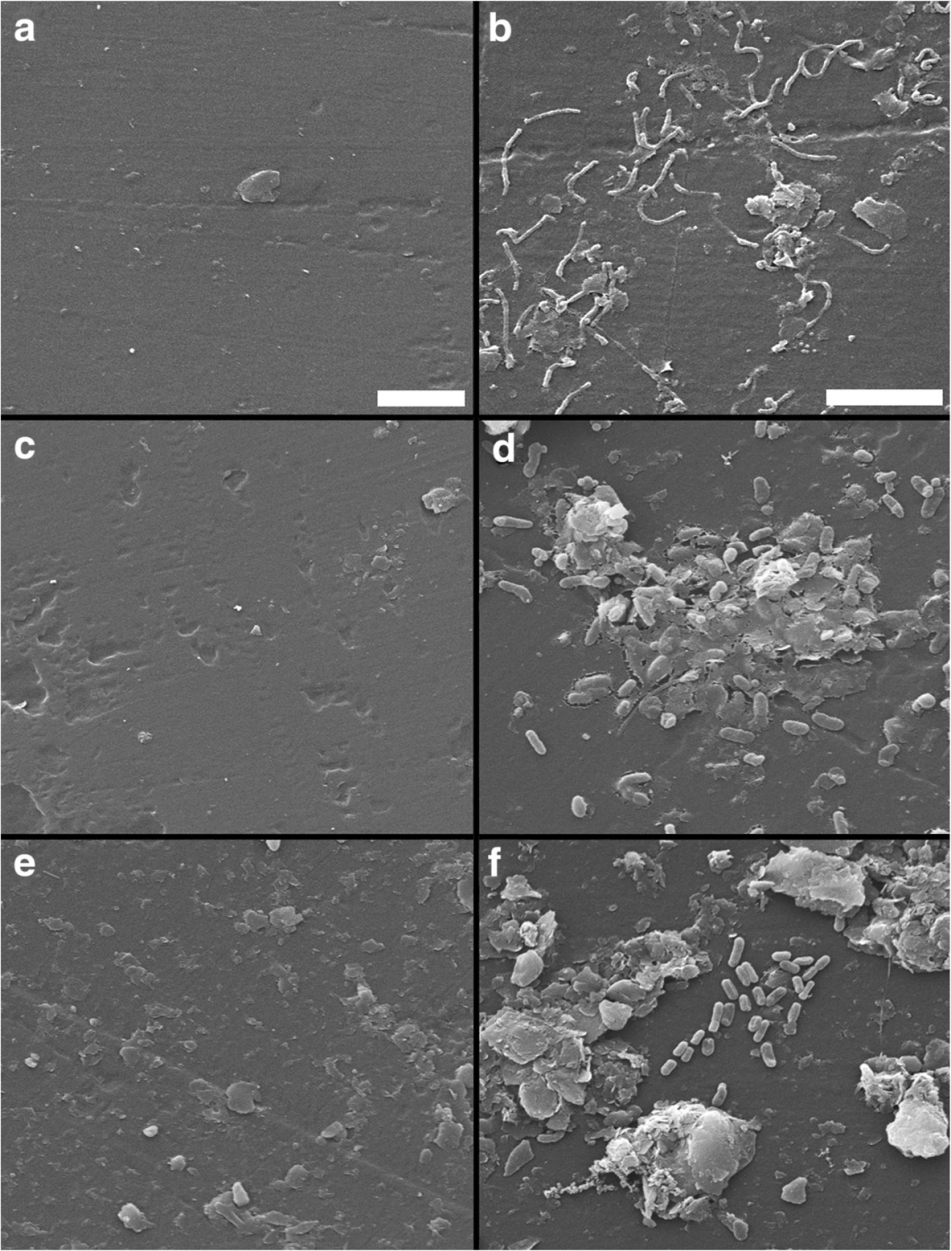
**Scanning electron micrographs showing prokaryotic attachment on LDPE microplastics.** Images were taken of LDPE fragments sampled from microcosm experiments containing coastal marine sediment from three sites: SP1 **(a and b)**, SP2 **(c and d)** and WB **(e and f)** at Spurn Point, UK, sampled either immediately or after 14 days (left-hand and right-hand panels, respectively). The scale bars are 5 μm.

**Figure 2:**
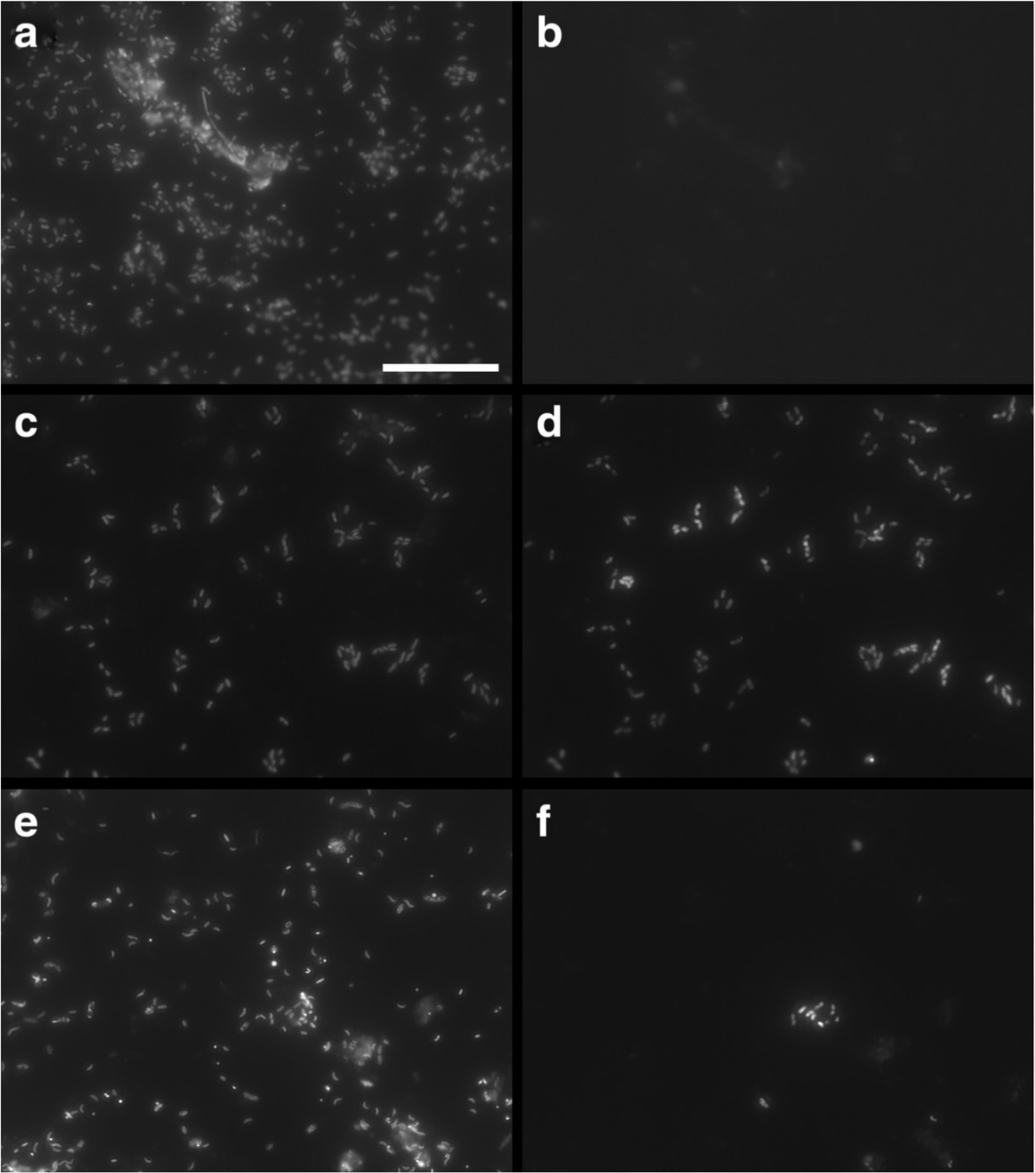
**Epifluorescence micrographs showing bacterial attachment onto LDPE microplastics.** The micrographs were obtained following 14 days of exposure to coastal sediment (sampling site SP2) in experimental microcosms. Micrographs corresponding to staining by 4′,6-diamidino-2-phenylindole (DAPI) are displayed in the left-hand panels **(a, c and e)**. Micrographs corresponding to staining by CARD-FISH are displayed on the right-hand panels, as shown for the oligonucleotide probes NON338 **(b)**, EUB338 I – III **(d)** and PSA184 **(f)**. The scale bar is 20 μm.

Scanning electron microscopy was used to examine microplastics obtained from sediment-LDPE microcosms either immediately or following 14 days of exposure in either sandy or silty sediment types collected from three sites (SP1, SP2 and WB) within the Humber Estuary, U.K. (see Additional files 1 and 2: Figure S1 and Table S1). Following detachment of loosely adhered material from the plastic surfaces by a gentle centrifugation step, attachment of morphologically diverse prokaryotic cells (rod- and spirilla-shaped) was observed on LDPE microplastics within all sediment types (Figure 1), with additional attachment by pennate diatoms (Additional file 3: Figure S2). Bacterial cells were found to be attached onto LDPE surfaces exposed to sediment from site SP2 for 14 days, as shown by 4′,6-diamidino-2-phenylindole (DAPI) staining and catalyzed reporter deposition *in situ* fluorescence hybridization (CARD-FISH) analysis performed with the oligonucleotide probes EUB338 I-III (Figure 2a-d). Bacteria typically constituted the majority of prokaryotic cells observed on the LDPE fragments (Figure 2d).

**Figure 3:**
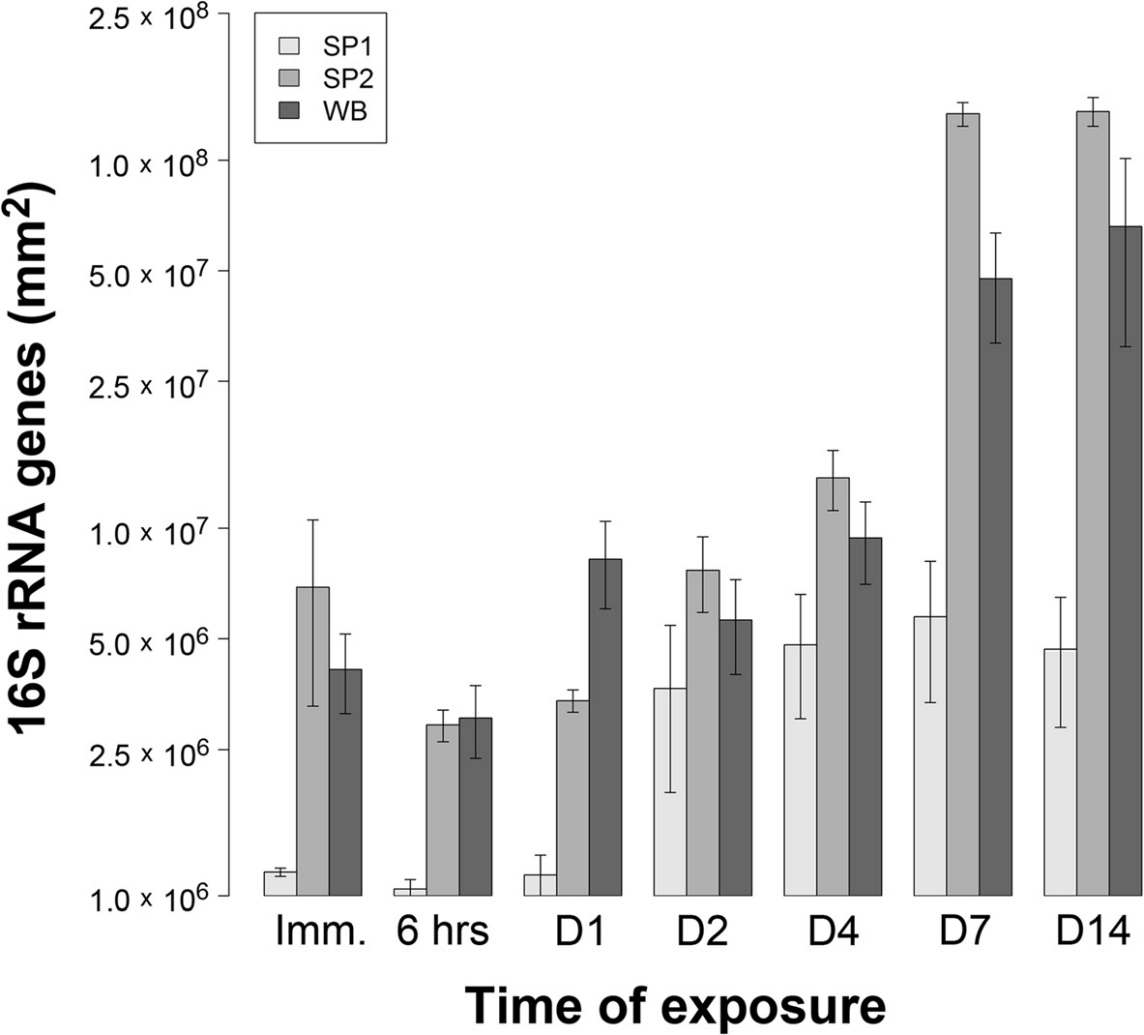
**Variation in the relative abundance of bacterial 16S rRNA genes.** The data are based on bacterial 16S rRNA genes amplified from DNA extracted from LDPE microplastics in sediments sampled over time. LDPE fragments were sampled in triplicate over time [immediately (Imm.), after 6 hours (hrs) and on days (D) 1, 2, 4, 7 and 14)] from microcosms containing sediments from three sites (SP1, SP2 and WB, as indicated). Abundances are expressed as 16S rRNA genes per mm^2^ of LDPE. Error bars represent one standard error (*n* = 3). Gene numbers were calculated from the following standard curve: *r* ^2^ = 0.979, *y* intercept = 42.6, slope = −4.64, *E* (amplification efficiency) = 64%, and *C*_t_ cut-off of 28.7.

Bacterial 16S rRNA gene numbers on LDPE fragments exposed to the three sediment types during the 14-day experiment were quantified (Figure 3) as a proxy of the relative abundance of plastic-colonizing bacteria using quantitative real-time PCR (Q-PCR). In order to obtain meaningful Q-PCR results, it is necessary to separate the DNA template amplification signal from background fluorescence [50,66]. Gene number estimates with mean *C*_t_ values less than 3.3 cycles lower than those corresponding to no-template controls (NTCs) (i.e., less than a log-fold difference in gene numbers) are potentially influenced by background interference [50]. For all three sites, this was the case for LDPE fragments sampled prior to Day 4 and additionally for site SP1 at Days, 4, 7 and 14. Consequently, estimates of bacterial 16S rRNA gene numbers for these samples were excluded from statistical analysis.

For LDPE microplastics retrieved from microcosms containing sediments from sites SP2 and WB, the abundance of 16S rRNA genes differed significantly both as a function of time of exposure to sediments and to sediment type (two-way ANOVA, *F*_2,12_ = 16.50, p < 0.001 and *F*_1,12_ = 14.65, p < 0.01, respectively). 16S rRNA gene numbers quantified following 7 and 14 days of exposure to sediments from sites SP2 and WB were approximately a log-fold higher than after four days of exposure (Tukey multiple comparison test for factor ‘exposure time’, p < 0.01 and p < 0.001 for sites SP2 and WB, respectively; Figure 3). There was no significant difference between numbers of 16S rRNA genes amplified from DNA extracted from LDPE microplastics sampled after 7 and 14 days of exposure to sediments. Following seven days of exposure to sediments, mean 16S rRNA gene numbers on LDPE surfaces corresponded to a ratio of 2.8:1.0 between sites SP2 ($\overline{x}$ = 1.3 × 10^8^ ± 9.9 × 10^6^ S.E.) and WB ($\overline{x}$ = 4.8 × 10^7^ ± 1.6 × 10^7^ S.E.) (Figure 3). For LDPE fragments exposed to sediments from site SP1, a nearly log-fold increase in the abundance of bacterial 16S rRNA genes on the plastic surfaces was observed over the duration of the experiment.

### The structure and diversity of LDPE-associated bacterial communities

**Figure 4:**
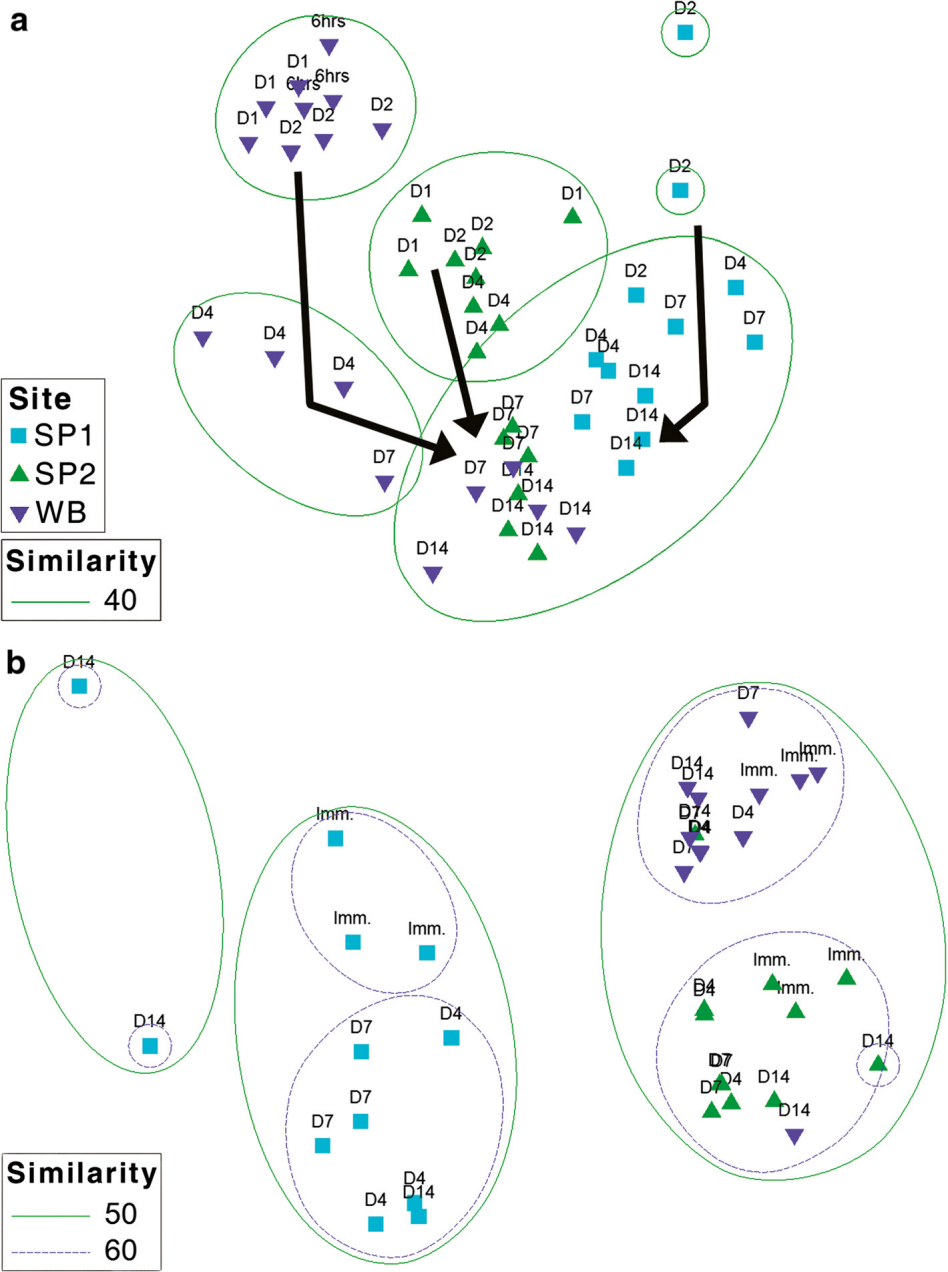
**Non**-**metric multidimensional scaling** (**nMDS**) **ordinations of sediment and LDPE microplastic**-**associated bacterial communities in sediment microcosms.** The ordinations were derived from a Bray-Curtis resemblance matrix calculated from square-root-transformed terminal restriction fragment (T-RF) relative abundance data. Data are shown for bacterial communities **a)** on LDPE fragments (stress = 0.16) and **b)** within sediments (stress = 0.13). Labels correspond to samples taken over time [immediately (Imm.), and after 6 hours, and on days (D) 1, 2, 4, 7 and 14)] from microcosms containing coastal marine sediments from three sites (SP1, SP2 and WB, as indicated). Similarity thresholds (%) are based on group-average clustering. Arrows indicate the temporal trajectory of bacterial community succession on LDPE surfaces.

Terminal-restriction fragment polymorphism (T-RFLP) data corresponding to LDPE microplastics exposed to sediments from site SP1 for less than two days, from site SP2 for less than one day and from site WB for less than six hours were excluded from statistical analysis due to low fluorescence signals. Bacterial communities present on LDPE microplastics differed significantly from those within the sediments, as demonstrated by T-RFLP analysis of 81 AluI-digested PCR products derived from DNA isolated from individual sediment-LDPE microcosms (two-way ANOSIM, global R = 0.71, p < 0.001; see Additional file 4: Figure S3). Initially (i.e., following two days of exposure to sediments), sediment type-specific communities were found on LDPE microplastics (one-way ANOSIM, global R = 0.67, p = 0.04; Figure 4a). Subsequently, significant variation was observed in the structure of the LDPE-associated bacterial communities during the 14-day experiment (Table 2). Specifically, there were significant shifts in the structure of LDPE-associated bacterial communities by Days 7 and 14 of the experiment, with notable convergence in the structure of these communities across the three sediment types (Figure 4a). One-way ANOSIM R declined from R = 0.67 (p < 0.001) to R = 0.01 (not significant), when comparing differences between LDPE-associated communities across different sediments at Day 2 vs. Day 14 and Day 7 vs. Day 14, respectively (Table 2). In contrast, sediment bacterial communities from each site remained significantly different from each other throughout the 14-day experiment (one-way ANOSIM, global R = 0.72, p < 0.001; Figure 4b). Moreover, no significant temporal variation was observed in the structure of the sediment bacterial communities (Table 2). Bacterial communities on LDPE microplastics became significantly less diverse over time (one-way ANOVA of Shannon’s diversity, *F* = 4.69, p = 0.008, *d.f. =* 3, 32) (Additional file 5: Table S2). In contrast, no significant temporal shifts were observed in the diversity of sediment bacterial communities (one-way ANOVA of Shannon’s diversity, *F* = 2.12, p = 0.117, *d.f. =* 3, 32) (Additional file 5: Table S2).

**Table 2:** **Pairwise comparisons (ANOSIM) of the structure of bacterial communities on microplastics and within sediments over time**

	**LDPE**-**microplastics**			**Sediment**		
Exposure time	2 days	4 days	7 days	Immediate	4 days	7 days
4 days	0.10 ^a^			0.08 (NS)		
7 days	0.46 ^a^	0.07 (NS)		0.12 (NS)	0.09 (NS)	
14 days	0.67 ^a^	0.38 ^a^	0.01 (NS)	0.12 (NS)	0.00 (NS)	0.03 (NS)

^a^p < 0.001 *NS* = not significant.

Global one-way ANOSIM R values for the factor ‘exposure time’ are derived from T-RFLP datasets generated following PCR amplification of bacterial 16S rRNA genes amplified from DNA isolated from sediment-LDPE microcosms from three sampling sites (SP1, SP2 and WB). Values are shown only for sampling intervals for which data were available for all three sites.

### The taxonomic identities of LDPE-associated bacterial genera

Clone libraries were constructed of PCR-amplified 16S rRNA genes from LDPE microplastics from sites SP1, SP2 and WB, following 14 days of exposure to either sandy or silty sediment types. A total of 251 sequences were generated across the three libraries. Rarefaction curves displayed a tendency for curvilinearity when using operational taxonomic unit (OTU) designations based on 95, 97 or 99% sequence similarity (Additional file 6: Figure S4). Moreover, Good’s coverage estimates of >75% were typically obtained for OTU designations at these levels of sequence identity (Additional file 7: Table S3).

**Figure 5:**
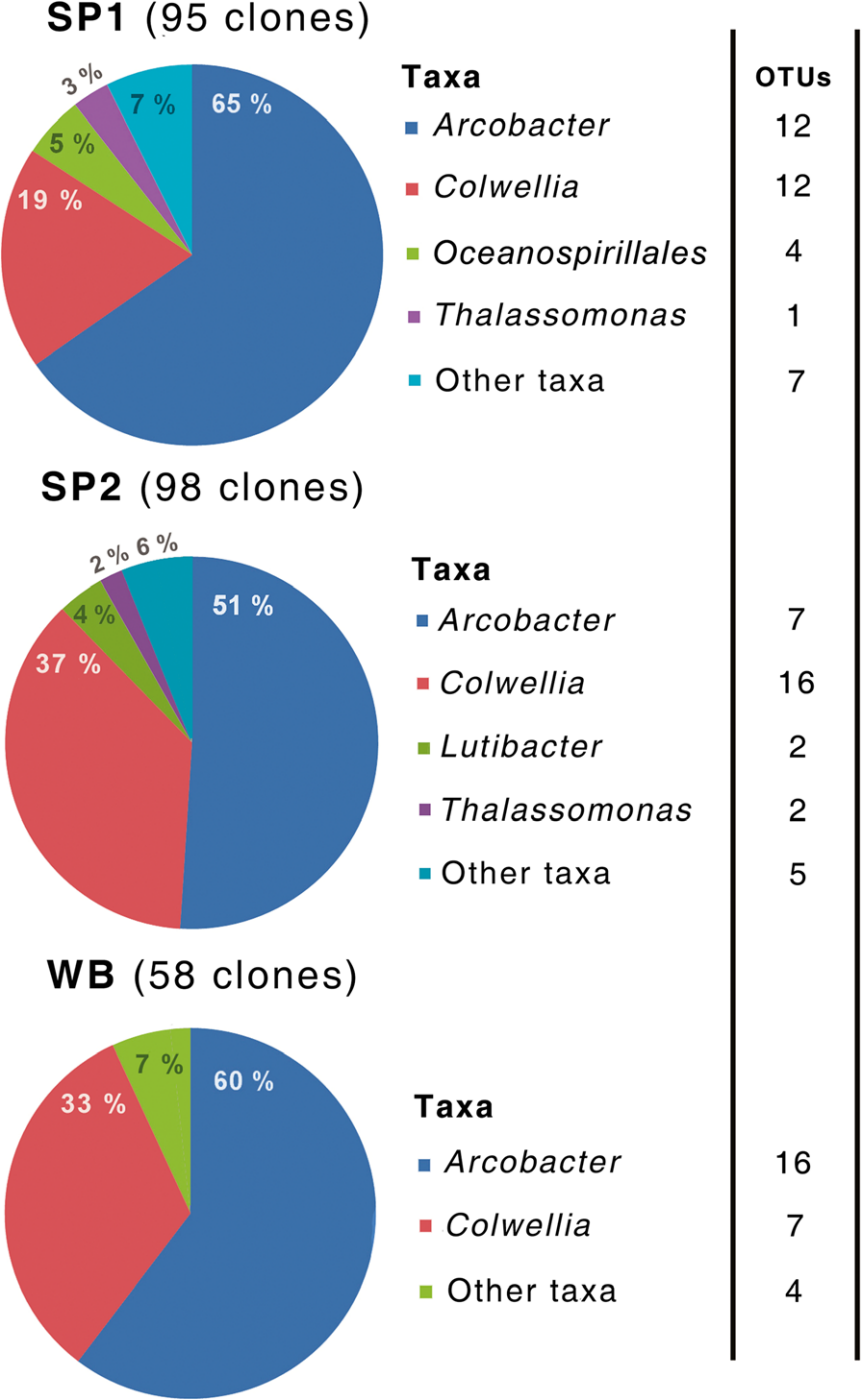
**Taxonomic composition and relative abundance** **(%)** **of LDPE microplastic**-**associated bacterial assemblages.** Clone libraries were generated following PCR amplification of bacterial 16S rRNA genes amplified from DNA isolated from LDPE fragments sampled after 14 days from sediment-LDPE microcosms from three sampling sites (SP1, SP2 and WB, as indicated). Clones were assigned to operational taxonomic units (OTUs) based on a similarity cut-off threshold of 99%, with numbers of individual OTUs within each taxon indicated.

**Figure 6:**
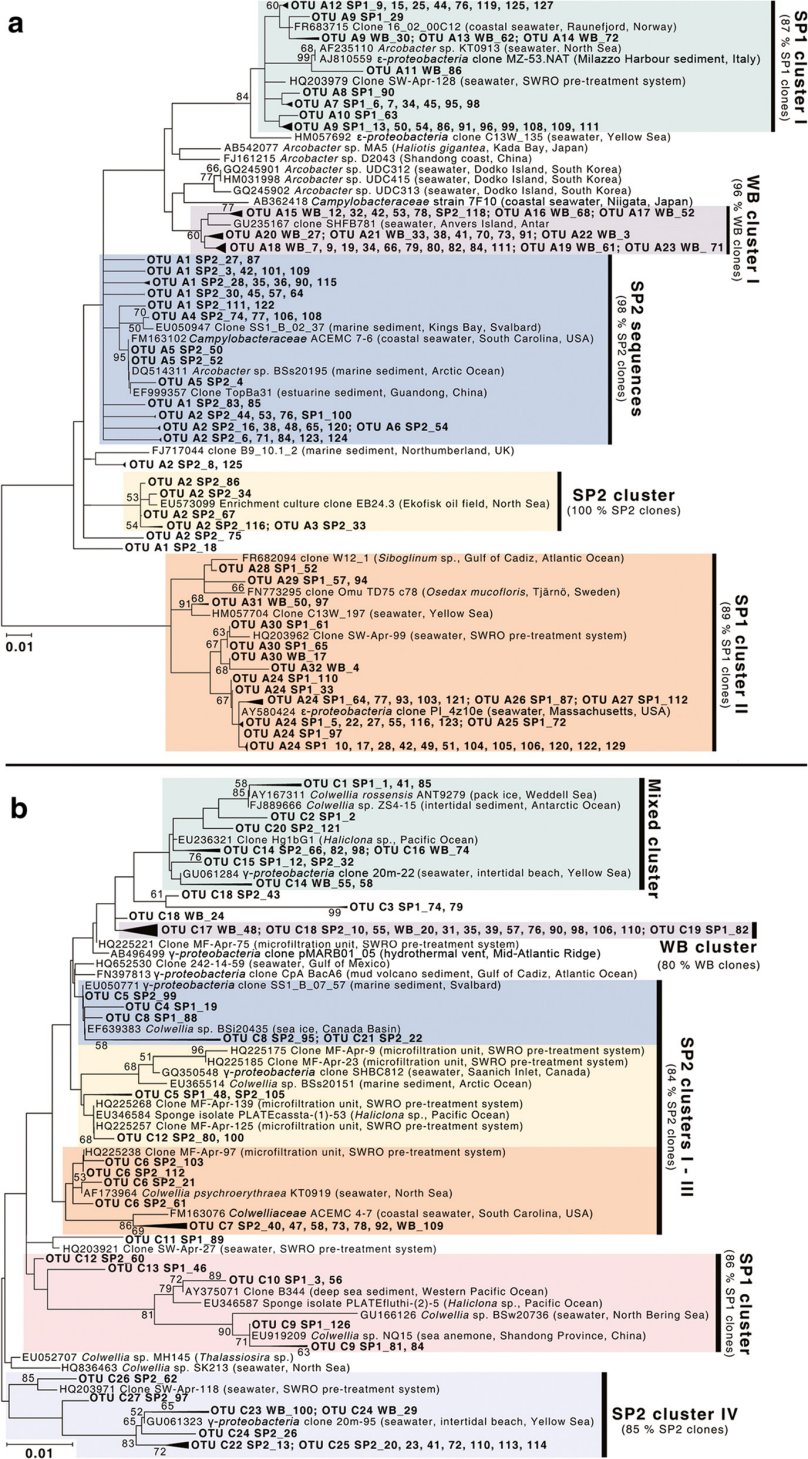
**Neighbor**-**joining phylogenetic trees showing taxonomic affiliation of LDPE**-**associated bacterial 16S rRNA gene sequences.** Data are shown for the genera **a)** *Arcobacter* and **b)** *Colwellia*. Sequences (in bold) were obtained from LDPE fragments sampled after 14 days from sediment-LDPE microcosms from three sites (SP1, SP2 and WB, as indicated). Most-closely related GenBank database sequences are included in the trees. Tree branches are collapsed according to OTU designations using a 99% similarity cut-off threshold. Highlighted regions indicate predominant sediment-specific populations. Bootstrap values of ≥ 50% are shown adjacent to nodes. The scale bar represents 1% sequence divergence.

16S rRNA gene sequences from the genera *Arcobacter* (*Epsilonproteobacteria*) and *Colwellia* (*Gammaproteobacteria*) were found to dominate the LDPE-associated bacterial assemblages, together comprising between 84 and 93% of sequences from the three sites (Figure 5). Neighbor-joining phylogenetic trees revealed a high degree of sediment-specific clustering within each genus, with 80 – 100% of the sequences within individual populations originating from a given sediment type (Figure 6). OTU-based analyses also showed sediment-specific clustering of these sequences, with 80 – 100% of the dominant OTUs within each genus typically originating from a single sampling site (Additional files 8 and 9: Figures S5 and S6). Moreover, the LDPE-affiliated communities from each site contained significantly different *Arcobacter* spp. populations, as assessed using LIBSHUFF in Mothur [60] (with Bonferroni correction, p < 0.05). Site-specific *Colwellia* spp. populations were also found. However, for this genus, differences between sites SP2 and WB were barely significant (p = 0.05), in agreement with the high similarities observed between the structures of the overall LDPE-associated bacterial communities at these two sites as determined by T-RFLP analysis (Figure 4).

The presence of *Colwellia* spp. on the surface of LDPE microplastics following 14 days of exposure to sediment from site SP2 was confirmed by CARD-FISH analysis using the oligonucleotide probe PSA184 (see Methods and Table 1) (Figure 2f). An attempt was also made to detect *Arcobacter* spp. on the LDPE surface using the probe ARC94 (Table 1). However, colonization of LDPE microplastics by *Arcobacter* spp. could not be visually demonstrated by CARD-FISH analysis due to the presence of non-specific fluorescence signals (data not shown).

## Discussion

In this study, a 14-day laboratory microcosm experiment has shown that bacteria present in coastal marine sediments can rapidly colonise low-density polyethylene (LDPE) microplastics. Scanning electron microscopy visually confirmed the direct attachment of primarily rod-shaped prokaryotic cells onto LDPE fragments within both sandy and silty sediment types (sampling sites SP1, SP2 and WB; see Additional file 2: Table S1), following detachment of loosely adhering materials from the plastic surfaces by centrifugation. Predominant attachment of bacteria onto LDPE surfaces was demonstrated by CARD-FISH analysis [33] (Figure 2). Moreover, log-fold increases in the abundance of 16S rRNA genes from LDPE-associated bacteria occurred within 7 days with 16S rRNA gene numbers differing significantly across sediment types, as shown by Q-PCR. Molecular analysis revealed that these LDPE-colonising bacterial communities were structurally and taxonomically distinct from those found in the surrounding sediment environment. T-RFLP analysis demonstrated significant time-dependent shifts in the structure of bacterial assemblages on LDPE microplastics, in particular, by Days 7 and 14 of the experiment, with successional convergence in microplastic bacterial community structure occurring between all three sediment types examined. In contrast, the structures of bacterial communities within sediments from the three sites remained significantly different from each other over the duration of the experiment, and no significant temporal patterns were observed in the structure of the sediment bacterial communities. Collectively, the present results provide several independent lines of evidence demonstrating the potential for site-specific bacterial colonization of LDPE microplastics in coastal sediments. For example, T-RFLP profiles of bacterial communities on LDPE fragments following less than two days of exposure to sediment from site SP1 typically exhibited low fluorescence intensities (data not shown), in agreement with the comparatively low 16S rRNA gene numbers estimated for this site (see Figure 3).

Despite recognition of the role and importance of sediments as a sink for the accumulation of plastic debris [7,9,12,16,17], previous research into the interactions between marine microorganisms and plastic debris has focused on investigation of microbial interactions with polymers in the water column [14,21,22,24-28]. While a prior study has reported a significant increase in the number of polyethylene-associated bacteria following three weeks of exposure in seawater [25] and the abundance of rod-shaped (Bacillus) bacteria on plastic surfaces in the North Pacific Gyre has recently been determined [27], the Q-PCR data reported herein provide the first quantitative evaluation of the potential for bacterial attachment onto LDPE fragments within coastal marine sediments. Moreover, prior culture-independent research into the bacterial colonization of marine plastic debris has only investigated communities following exposure of polymers in seawater for up to 72 hours, representing early colonization events [21,22,26]. Our research has demonstrated that over longer time periods (up to 14 days), successional shifts in the structure of LDPE-microplastic bacterial assemblages occur, highlighting the need to undertake analyses over varying timescales to fully understand microbial biofilm colonization processes on microplastics [23].

By the end of the 14-day experiment, LDPE microplastics were primarily colonized by location-specific populations of *Arcobacter* spp. (*Epsilonproteobacteria*) and *Colwellia* spp. (*Gammaproteobacteria*), as shown by 16S rRNA gene sequencing. It is possible that loosely adhered bacteria may have been removed from the microplastics during the initial washing step (see Methods) during recovery from sediments. Nevertheless, whilst the occurrence and identity of rarer taxa on the LDPE surface may be underestimated, these communities were clearly dominated by *Arcobacter* and *Colwellia* spp. Notably, neither taxon was found to be present upon polyethylene or polypropylene fragments in open ocean water (27). Direct attachment by *Colwellia* spp. onto the surface of LDPE fragments was additionally confirmed by CARD-FISH. Both rarefaction analysis and Good’s coverage estimates for operational taxonomic units (OTUs) supported majority representation of the overall taxon diversity (eg. >82% for 97% OTU designation) within the plastic-associated bacterial communities (see Additional files 6 and 7: Figure S4 and Table S3). Interestingly, previous studies characterising bacterial-plastic interactions within U.S.A., Chinese and Korean coastal waters identified *Roseobacter* spp. and other *Alphaproteobacteria* as the primary colonists of acryl, polyurethane, poly(methyl methacrylate) and polyvinylchloride surfaces within 24 hours of exposure [21,22,26]. In contrast, *Epsilonproteobacteria* were not detected on any of these polymer types. Moreover, the relative abundance of *Gammaproteobacteria* found attached to polymers in seawater was repeatedly found to significantly decrease after 24 hours of exposure [21,22,26]. Although *Vibrio* spp. have recently been found to constitute up to 24% of the bacterial OTUs discovered on a polypropylene sample from the North Atlantic Ocean [28], members of this genus were not detected on microplastic fragments as part of the present study. These differences in taxon composition between bacterial assemblages colonising polymers within either sediment or water may be attributable to several factors, including variation in the structure, composition and activities of bacterial communities between environmental compartments (i.e., sediment versus water), differences in experimental conditions, polymer types and durations of exposure [21-23,26-28,67]. It must also be recognised that laboratory-based microcosm experiments can be subject to inherent biases [68] that may have influenced the taxonomic identities of the plastic-colonizing bacteria reported in our study. Despite these uncertainties, the present data suggest that distinct plastisphere habitats are likely to occur within diverse types of coastal sediments, in addition to those previously discovered within the marine water column [28].

Whilst the LDPE-affiliated *Arcobacter* 16S rRNA gene sequences reported in this study were closely related to those from isolates and/or clones from marine environments (Figure 4), the ecological role(s) of this genus are poorly understood. Although *Arcobacter* spp. are increasingly found within marine environments including coastal habitats and sediments [69-71], prior research has primarily focused on their role as clinical and animal pathogens (reviewed in [71]). However, Assanta et al. [29] have demonstrated attachment of *Arcobacter butzleri* onto polyethylene pipe surfaces used in water distribution systems. *Arcobacter* spp. have also been found to colonise carbon steel surfaces in seawater near Qingdao, China [72]. *Colwellia* spp. identified in this study were most closely related to those in both polar and sub-tropical marine habitats (Figure 6). Interestingly, whilst C*olwellia* are considered as psychrophilic and have predominantly been found within polar environments [73], members of this genus have also been identified as minor components of both acryl- and steel-colonizing bacterial assemblages within coastal waters near Korea and China, respectively [26,72]. Furthermore, *Colwellia* spp. are known to produce extracellular polymeric substances [74] that may enhance biofilm formation on plastic surfaces. While research into the direct detection of extracellular polymeric substances and other bacterial metabolites on LDPE fragments was beyond the scope of our investigation, bacterial-surface interactions during primary colonization are known to exert a significant influence on the composition and further successional recruitment of microorganisms on plastic surfaces [21,22].

Although the ecological roles of the LDPE-colonising bacteria described in this study are unknown, both *Arcobacter* and *Colwellia* are additionally present in hydrocarbon-rich environments, with both genera having previously been affiliated with hydrocarbon contaminant mineralisation in cold ecosystems [75-81]. Interestingly, several hydrocarbon-degrading microbial taxa have also been found to associate with plastic debris in seawater [28]. To advance our understanding of microbial-plastic interactions and their implications for research into the environmental impacts and/or fate of plastic litter in the marine environment, more work is needed to characterize the ability of microplastic-associated bacteria to mediate breakdown of plastic co-contaminants, additives and/or of the petroleum hydrocarbon-derived polymers themselves [23]. Wider investigation is also required in order to determine whether the structure, taxonomic identities and metabolic functions of plastic-affiliated microbial consortia vary across different polymers, *in situ* environmental conditions and plastic fragments retrieved from benthic and pelagic habitats [21,22,26,67].

## Conclusion

In summary, we have used a microcosm experiment that demonstrates the capacity for rapid attachment of microorganisms onto LDPE microplastics within coastal marine sediments. We found that the structure of the bacterial communities present on LDPE surfaces converged rapidly, with these plastisphere communities dominated after 7 to 14 days by members of two bacterial genera (*Arcobacter* and *Colwellia*), despite the bacterial communities within the three sediments being highly divergent from each other. Whilst (all) microcosm experiments can only provide an abstraction of the complex ecology of a natural environmental system, to our knowledge, this study nevertheless represents the first quantitative and culture-independent assessment of the potential for microplastics in marine sediments to function as sites for microbial colonization and biofilm formation. Although the metabolic activities of the LDPE-associated bacterial assemblages reported in this study are unknown, our data suggest that potentially hydrocarbonoclastic bacterial taxa can be found not only on plastic fragments within the water column [28], but within sediments as well. As such, the present results provide a starting point for research into the formation, ecology and functions of plastisphere-associated microbial assemblages in benthic marine habitats, and understanding how they influence the environmental impacts and fate of microplastic pollution within sediment systems. A major future challenge of such research will be to investigate the dynamic successional changes and ecological interactions occurring within microbial communities on plastic fragments, during transport from freshwater into marine systems, and additionally, from the water column into sediment systems. For example, recent research has highlighted differences in microbial community structure within the plastisphere between different freshwater environments [82]. The colonization of plastic by microorganisms can additionally be considered as biofouling, with such biofilm formation suggested to contribute towards changes in the buoyant density of polymers, leading to transport of plastic from the oceanic surface waters into the deeper water column and into sediments [83]. Complicating matters further, the plastisphere has been shown to host a diverse assemblage of microbial eukaryotes and invertebrates [84], and the structure of plastisphere communities varies both between different polymer types and between different seasons [85]. Additionally, there is a need to identify those microorganisms that are preferentially able to colonize and interact with plastic surfaces, as opposed to generalists that can colonize other surfaces (e.g., glass or metal) in the aquatic environment [21,28,85]. We are clearly still at the very early stages of describing and understanding the ecology and biodiversity of the plastisphere, and unravelling these systems will require the characterization of plastic fragments isolated across diverse environments, as well as exposure experiments that can effectively mimic the complex journeys of plastic pollutants within aquatic ecosystems.

### Availability of supporting data

16S rRNA gene sequences supporting the results of this article are available in the GenBank Database (https://www.ncbi.nlm.nih.gov/genbank/) under accession numbers JF928573 to JF928823.

## Additional files

Additional file 1: Figure S1. Locations of sediment sampling sites. Locations of field sites used for sediment sampling at Spurn Point, Yorkshire, UK. The regional location of the sampling sites within the UK. is shown in the inset together with eastern latitude and northern longitude (°), as indicated. Additional file 2: Table S1. Particle size distribution and loss on ignition data. Particle size distribution (PSD) and loss on ignition (LOI) for coastal marine sediments from three sites (SP1, SP2 and WB) at Spurn Point, UK. Values are given in duplicate for the <1-mm fraction. The dominant particle size fraction for each sediment is highlighted in bold. Additional file 3: Figure S2. SEM image showing a pennate diatom attached to LDPE. Scanning electron microscope image showing attachment by prokaryotic cells and an unidentified pennate diatom onto the LDPE-microplastic surface. Prokaryotic cells that appear to be embedded within the polymer matrix are indicated by arrows. The image is of LDPE sampled after 14 days from a microcosm experiment containing coastal marine sediment from Spurn Point, UK. (site SP2). The scale bar is 5 μm. Additional file 4: Figure S3. Representative T-RFLP electropherograms. Representative T-RFLP electropherograms of bacterial communities in A) coastal marine sediments and B) within the LDPE ‘plastisphere’. T-RFLP profiles were generated following PCR amplification of bacterial 16S rRNA genes amplified from DNA isolated from sediment-LDPE microcosms from three sampling sites (SP1, SP2 and WB, as indicated), sampled after 14 days. Additional file 5: Table S2. Shannon’s diversity indices (*H*’) for bacterial assemblages. Shannon’s diversity indices (*H*’) for bacterial assemblages within sediments and the LDPE plastisphere over time. The values are derived from T-RFLP datasets generated following PCR amplification of bacterial 16S rRNA genes amplified from DNA isolated from sediment-LDPE microcosms from three sampling sites (SP1, SP2 and WB). The data are given as mean ± S.E (*n* = 3). Additional file 6: Figure S4. Rarefaction curves for 16S rRNA gene clone libraries. Rarefaction curves for bacterial 16S rRNA gene clone libraries of LDPE- plastisphere assemblages. Clone libraries were generated following PCR amplification of bacterial 16S rRNA genes amplified from DNA isolated from sediment-LDPE microcosms from three sampling sites (SP1, SP2 and WB, as indicated), sampled after 14 days. Rarefaction curves are shown for operational taxonomic unit (OTU) designations for unique sequences and for OTUs based on similarity cut-off thresholds ranging from 99 to 95%, following removal of chimeric sequences. The dashed lines represent 95% confidence intervals. Additional file 7: Table S3. Good’s coverage estimates for 16S rRNA gene clone libraries. Good’s coverage estimates for 16S rRNA gene clone libraries. Values are given for operational taxonomic unit (OTU) designations for unique sequences and for OTUs based on similarity cut-off thresholds ranging from 99 to 95%, following removal of chimeric sequences. 16S rRNA gene clone libraries were generated from the LDPE plastisphere following 14-day laboratory microcosm experiments in coastal marine sediments from three sites (SP1, SP2 and WB). Additional file 8: Figure S5. Relative contributions (%) of OTUs on microplastics. Heat maps displaying the overall relative contributions (%) of the most dominant bacterial operational taxonomic units (OTUs) within the LDPE-sediment interface. Data are shown for the genera a) *Arcobacter* and b) *Colwellia*, with the contributions (%) of each OTU shown beneath each OTU label. Sequences were obtained from LDPE microplastics sampled after 14 days from sediment-LDPE microcosms from three sites (SP1, SP2 and WB, as indicated). Additional file 9: Figure S6. Frequencies of OTUs on microplastics. Frequencies of LDPE plastisphere *Arcobacter*- and *Colwellia*-affiliated operational taxonomic units (OTUs) within and across different sediment sampling sites. Values are shown for OTUs based on similarity cut-off thresholds of 99% (A and B), 97% (C and D) and 95% (E & F). Clone libraries were generated following PCR amplification of bacterial 16S rRNA genes amplified from DNA isolated from LDPE microplastics sampled after 14 days from sediment-LDPE microcosms from three sampling sites (SP1, SP2 and WB, as indicated).
