# Supplementary figures and images for: Rapid bacterial colonization of low-density polyethylene microplastics in coastal sediment microcosms
Source: BMC Microbiol. 2014 Sep 23;14:232. doi: 10.1186/s12866-014-0232-4 (PMC4177575; doi:10.1186/s12866-014-0232-4)

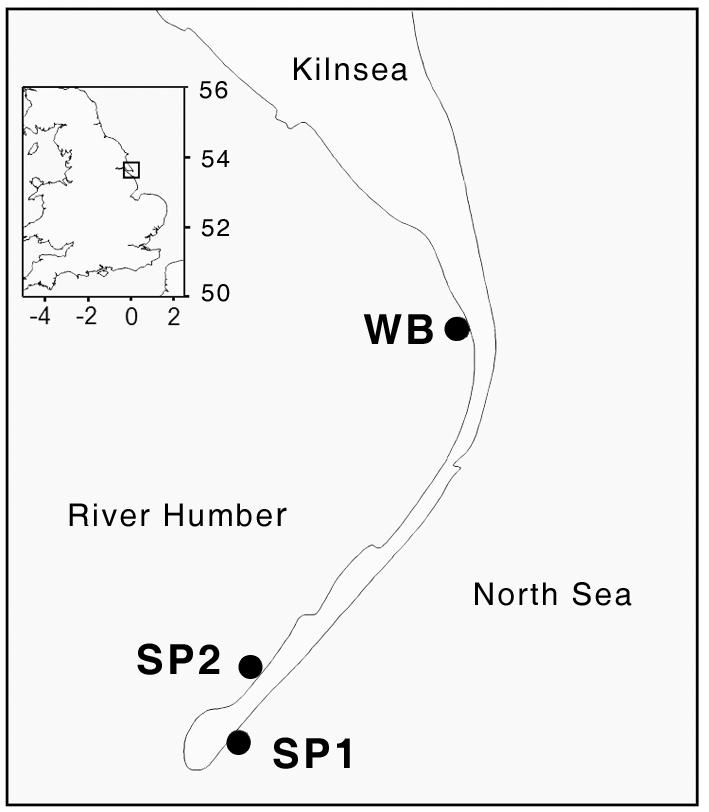

Supplement: Additional file 1: Figure S1. — Locations of sediment sampling sites. Locations of field sites used for sediment sampling at Spurn Point, Yorkshire, UK. The regional location of the sampling sites within the UK. is shown in the inset together with eastern latitude and northern longitude (°), as indicated. [file 12866_2014_232_MOESM1_ESM.tiff]

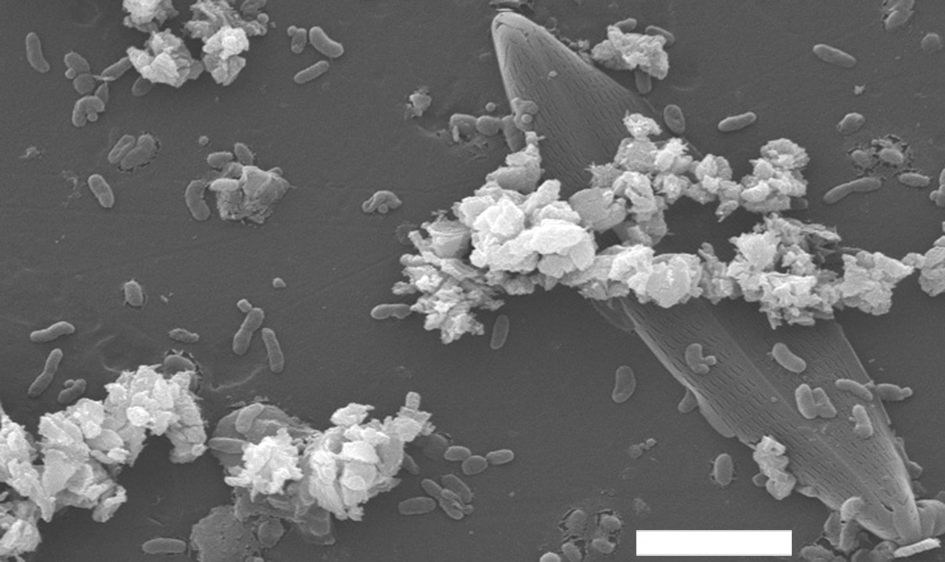

Supplement: Additional file 3: Figure S2. — SEM image showing a pennate diatom attached to LDPE. Scanning electron microscope image showing attachment by prokaryotic cells and an unidentified pennate diatom onto the LDPE-microplastic surface. Prokaryotic cells that appear to be embedded within the polymer matrix are indicated by arrows. The image is of LDPE sampled after 14 days from a microcosm experiment containing coastal marine sediment from Spurn Point, UK. (site SP2). The scale bar is 5 μm. [file 12866_2014_232_MOESM3_ESM.tiff]

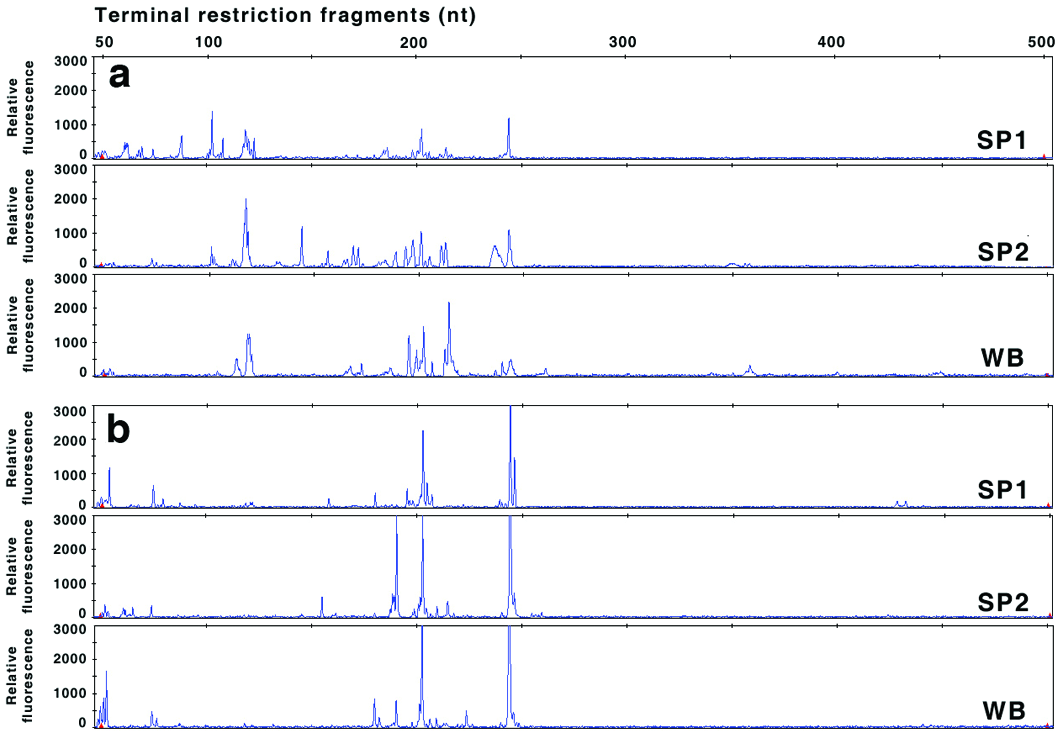

Supplement: Additional file 4: Figure S3. — Representative T-RFLP electropherograms. Representative T-RFLP electropherograms of bacterial communities in A) coastal marine sediments and B) within the LDPE ‘plastisphere’. T-RFLP profiles were generated following PCR amplification of bacterial 16S rRNA genes amplified from DNA isolated from sediment-LDPE microcosms from three sampling sites (SP1, SP2 and WB, as indicated), sampled after 14 days. [file 12866_2014_232_MOESM4_ESM.tiff]

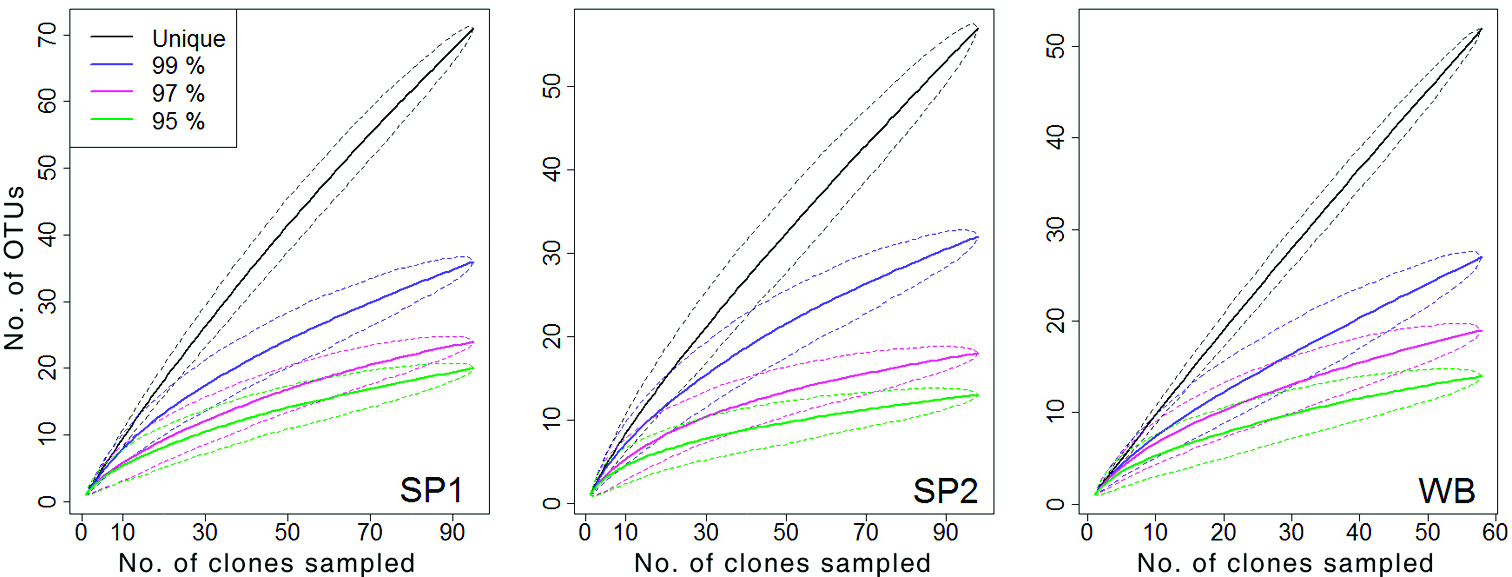

Supplement: Additional file 6: Figure S4. — Rarefaction curves for 16S rRNA gene clone libraries. Rarefaction curves for bacterial 16S rRNA gene clone libraries of LDPE- plastisphere assemblages. Clone libraries were generated following PCR amplification of bacterial 16S rRNA genes amplified from DNA isolated from sediment-LDPE microcosms from three sampling sites (SP1, SP2 and WB, as indicated), sampled after 14 days. Rarefaction curves are shown for operational taxonomic unit (OTU) designations for unique sequences and for OTUs based on similarity cut-off thresholds ranging from 99 to 95%, following removal of chimeric sequences. The dashed lines represent 95% confidence intervals. [file 12866_2014_232_MOESM6_ESM.tiff]

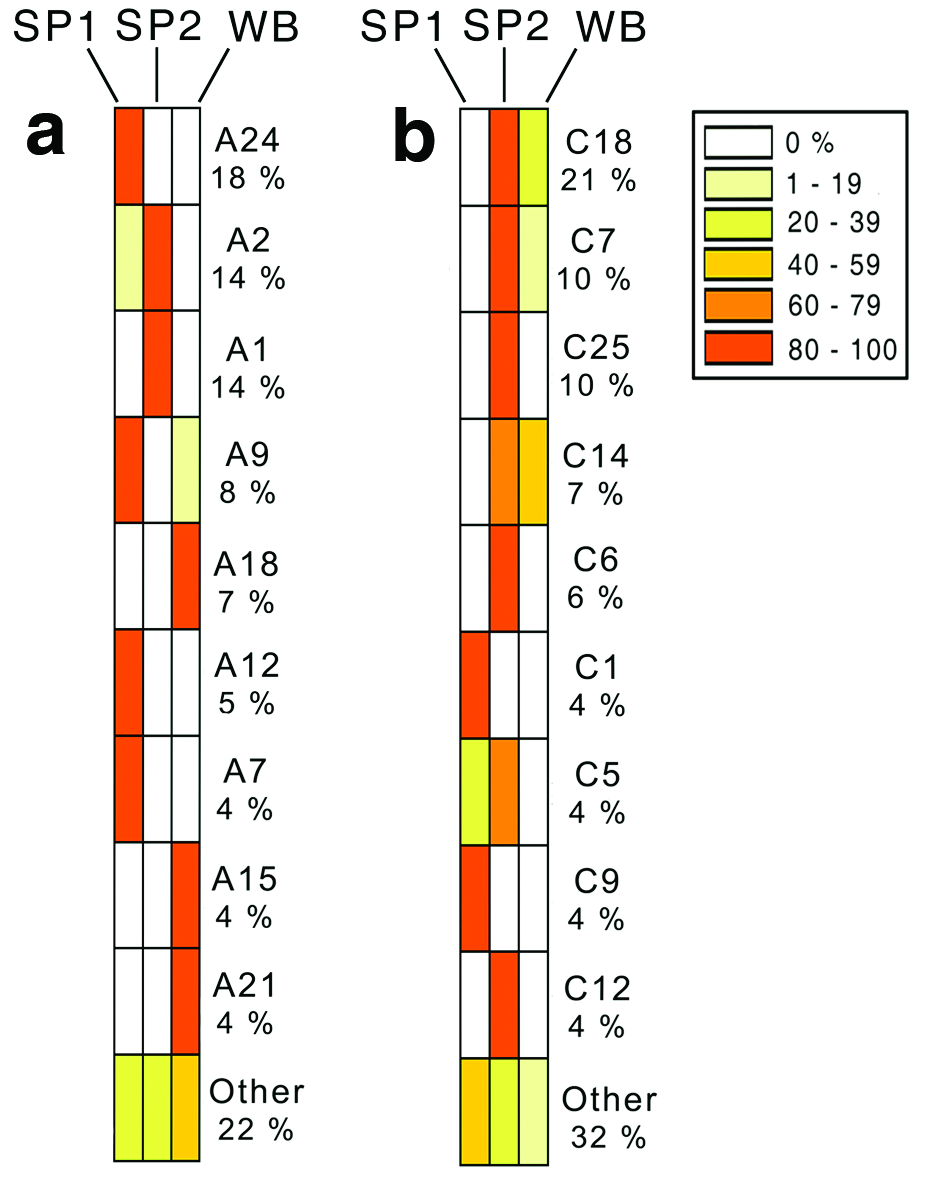

Supplement: Additional file 8: Figure S5. — Relative contributions (%) of OTUs on microplastics. Heat maps displaying the overall relative contributions (%) of the most dominant bacterial operational taxonomic units (OTUs) within the LDPE-sediment interface. Data are shown for the genera a) Arcobacter and b) Colwellia, with the contributions (%) of each OTU shown beneath each OTU label. Sequences were obtained from LDPE microplastics sampled after 14 days from sediment-LDPE microcosms from three sites (SP1, SP2 and WB, as indicated). [file 12866_2014_232_MOESM8_ESM.tiff]

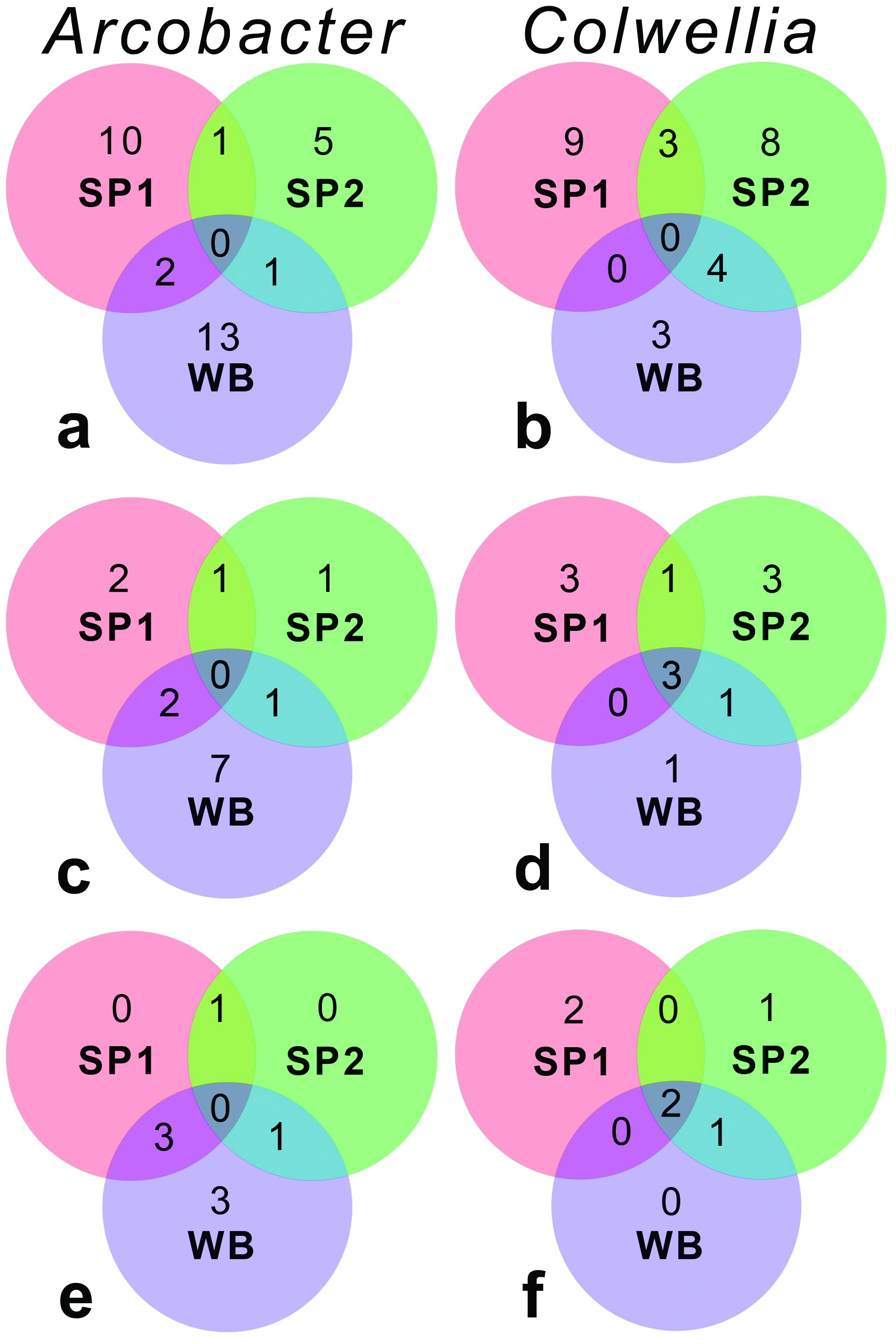

Supplement: Additional file 9: Figure S6. — Frequencies of OTUs on microplastics. Frequencies of LDPE plastisphere Arcobacter- and Colwellia-affiliated operational taxonomic units (OTUs) within and across different sediment sampling sites. Values are shown for OTUs based on similarity cut-off thresholds of 99% (A and B), 97% (C and D) and 95% (E & F). Clone libraries were generated following PCR amplification of bacterial 16S rRNA genes amplified from DNA isolated from LDPE microplastics sampled after 14 days from sediment-LDPE microcosms from three sampling sites (SP1, SP2 and WB, as indicated). [file 12866_2014_232_MOESM9_ESM.tiff]
