# Supplementary material for: Rapid bacterial colonization of low-density polyethylene microplastics in coastal sediment microcosms
Source: BMC Microbiol. 2014 Sep 23;14:232. doi: 10.1186/s12866-014-0232-4 (PMC4177575; doi:10.1186/s12866-014-0232-4)
Supplement: Additional file 2: Table S1. — Particle size distribution and loss on ignition data. Particle size distribution (PSD) and loss on ignition (LOI) for coastal marine sediments from three sites (SP1, SP2 and WB) at Spurn Point, UK. Values are given in duplicate for the <1-mm fraction. The dominant particle size fraction for each sediment is highlighted in bold. [file 12866_2014_232_MOESM2_ESM.docx]

|  | **Sieved fractions (%)** | |  | **<1 mm fraction (%)** | | | | |  |  |
| --- | --- | --- | --- | --- | --- | --- | --- | --- | --- | --- |
| **Site** | > 2 mm | 1 – 2 mm |  | Coarse sand | Medium sand | Fine sand | Silt | Clay |  | **LOI (%)** |
| SP1 | 1.8 | 2.1 |  | 1.8 / 2.3 | 31.1 / 31.6 | **66.8 / 65.8** | 0.4 / 0.3 | Absent |  | 1.0 |
| SP2 | 1.4 | 1.0 |  | 24.4 / 21.7 | **65.1 / 64.7** | 10.2 / 13.4 | 0.3 / 0.2 | Absent |  | 1.7 |
| WB | Absent | Absent |  | 0.5 / 0.6 | 9.5 / 9.8 | 24.8 / 25.6 | **57.0 / 59.6** | 8.1 / 4.4 |  | 10.1 |
