# Supplementary material for: Rapid bacterial colonization of low-density polyethylene microplastics in coastal sediment microcosms
Source: BMC Microbiol. 2014 Sep 23;14:232. doi: 10.1186/s12866-014-0232-4 (PMC4177575; doi:10.1186/s12866-014-0232-4)
Supplement: Additional file 5: Table S2. — Shannon’s diversity indices (H’) for bacterial assemblages. Shannon’s diversity indices (H’) for bacterial assemblages within sediments and the LDPE plastisphere over time. The values are derived from T-RFLP datasets generated following PCR amplification of bacterial 16S rRNA genes amplified from DNA isolated from sediment-LDPE microcosms from three sampling sites (SP1, SP2 and WB). The data are given as mean ± S.E (n = 3). [file 12866_2014_232_MOESM5_ESM.docx]

|  | **Sampling interval** | | | | |
| --- | --- | --- | --- | --- | --- |
| **Substrate** | Immediate | 2 days | 4 days | 7 days | 14 days |
| Sediment | 3.41 ± 0.05 | N/A ** | 3.23 ± 0.07 | 3.26 ± 0.05 | 3.31 ± 0.04 |
| Plastisphere | N/A * | 3.26 ± 0.11 | 3.05 ± 0.10 | 2.88 ± 0.09 | 2.80 ± 0.08 |

***** Data not available for all sampling sites ** No samples collected
