# Supplementary material for: Rapid bacterial colonization of low-density polyethylene microplastics in coastal sediment microcosms
Source: BMC Microbiol. 2014 Sep 23;14:232. doi: 10.1186/s12866-014-0232-4 (PMC4177575; doi:10.1186/s12866-014-0232-4)
Supplement: Additional file 7: Table S3. — Good’s coverage estimates for 16S rRNA gene clone libraries. Good’s coverage estimates for 16S rRNA gene clone libraries. Values are given for operational taxonomic unit (OTU) designations for unique sequences and for OTUs based on similarity cut-off thresholds ranging from 99 to 95%, following removal of chimeric sequences. 16S rRNA gene clone libraries were generated from the LDPE plastisphere following 14-day laboratory microcosm experiments in coastal marine sediments from three sites (SP1, SP2 and WB). [file 12866_2014_232_MOESM7_ESM.docx]

|  |  | **Coverage estimates (%) for different OTU designations** | | | |
| --- | --- | --- | --- | --- | --- |
| **Site** | **Number of clones** | Unique sequences | 99 % | 97 % | 95 % |
| SP1 | 95 | 38.9 | 76.8 | 87.4 | 88.4 |
| SP2 | 98 | 51.0 | 81.6 | 91.8 | 93.9 |
| WB | 58 | 15.5 | 63.8 | 82.8 | 87.9 |
